# Determinants of a GP visit and cervical cancer screening examination in Great Britain

**DOI:** 10.1371/journal.pone.0174363

**Published:** 2017-04-05

**Authors:** Alexander Michael Labeit, Frank Peinemann

**Affiliations:** 1 University of Sheffield, School of Health and Related Sciences, Sheffield, United Kingdom; 2 FOM University of Applied Science for Economics & Management, Essen, Germany; 3 Children's Hospital, University Hospital of Cologne, Cologne, Germany; University of Westminster, UNITED KINGDOM

## Abstract

**Objective:**

In the UK, women are requested to attend a cervical cancer test every 3 years as part of the NHS Cervical Screening Programme. This analysis compares the determinants of a cervical cancer screening examination with the determinants of a GP visit in the same year and investigates if cervical cancer screening participation is more likely for women who visit their GP.

**Methods:**

A recursive probit model was used to analyse the determinants of GP visits and cervical cancer screening examinations. GP visits were considered to be endogenous in the cervical cancer screening examination. The analysed sample consisted of 52,551 observations from 8,386 women of the British Household Panel Survey.

**Results:**

The analysis showed that a higher education level and a worsening self-perceived health status increased the probability of a GP visit, whereas smoking decreased the probability of a GP visit. GP visits enhanced the uptake of a cervical cancer screening examination in the same period. The only variables which had the same positive effect on both dependent variables were higher education and living with a partner. The probability of a cervical cancer screening examination increased also with previous cervical cancer screening examinations and being in the recommended age groups. All other variables had different results for the uptake of a GP visit or a cervical cancer screening examination.

**Conclusions:**

Most of the determinants of visiting a GP and cervical cancer screening examination differ from each other and a GP visit enhances the uptake of a smear test.

## Introduction

About 3000 women receive a cervical cancer diagnosis and 1000 women die from cervical cancer every year in the UK [1]. Cervical cancer can be prevented if abnormalities are detected at an early stage. Therefore, women are encouraged to attend cervical cancer screening examinations within the Cervical Screening Programme (NHSCSP) which are offered by the National Health Service (NHS) which is the national public health service of the UK. A high participation rate is especially important for the cervical cancer screening examination, because the smear test gives the possibility of early cervical cancer detection. Cervical cancer is one of the cancer types which has a high chance of cure if it is detected in an early stage [2].

The NHS Cervical Screening Programme (NHSCSP) is a nationally organised prevention programme which offers women a smear test in certain time intervals. The smear test takes a small sample of cells from the cervix and the sample is analysed under a microscope to detect abnormalities. The cervical cancer screening examination can be done in a GP practice or appropriate service institution such as a woman or family clinic or the genito-urinary medicine (GUM) department of a hospital [3]. The NHSCSP defines rules about the age of first invitation and screening intervals [4]. The NHSCSP has a different age of first invitation and screening intervals in England, Scotland and Wales, because the NHS England, NHS Scotland and the NHS Wales are responsible for the management of the programme in their countries. The invitation is dependent on age and screening examinations in the previous years [1]. The age of the first invitation depends in which part a woman lives in Great Britain: it is age 25 in England since 2003 and 20 in Scotland, Wales and in England before 2003 [5, 6]. Before 2003 there was a 3 to 5 yearly recall period between the age of the first invitation and age 49. The local Primary Care Trust was responsible for the invitation policy [7]. A majority of 85% of Primary Care Trusts invited women for a cervical cancer screening every 3 years and a minority of 15% had a mixed policy of inviting every 3 or 5 years depending on the age of women until 2002 [8]. After 2003 the policy changed to a 3 yearly recall period in all parts of Great Britain. Women aged 50 and over are invited to cervical cancer screening examinations every three years until age 60 in Scotland, until age 64 in Wales, and every 5 years in England until 64 [5]. No invitation letters will be sent to women over age 60 in Scotland and over age 65 in England and Wales. Invitation letters will be sent only in the case of a previous abnormal test result.

Economic models for the demand of health care in general [9] and for preventative medical care in particular [10] are based on human capital models. These models consider education, training, and investment in health care as the most important investments in human capital and investments in human capital raise the productivity of individuals including the production of health. The Grossman model of health production considers each individual as both a consumer and a producer of health. Health is treated as a stock variable which depreciates and so it is necessary to investment in health to maintain the stock. This model explains how age, health status, education, and income have an influence on the production of health and this model can also be used to explain how these variables influence the demand of a GP visit and a cervical cancer screening examination [9]. The Grossman model makes the distinction between acute and preventative care, however no uncertainty is considered in this model [11]. Acute care is relevant for the consumption aspect of health, whereas preventative care is relevant for the investment aspect. Other models take only uncertainty into consideration and no distinction is made between acute and preventative care [12]. Therefore, one potential disadvantage of these models is that both aspects are not considered at the same time in detail: the distinction between acute and preventative health care and uncertainty. Only one economic model explicitly considers the demand for preventative health care and uncertainty in a stochastic dynamic model [13].

Age can have different effects on the demand for a GP visit and a cervical cancer screening examination [10]. On one hand, health depreciates with an increasing rate at older ages. One explanation is the higher prevalence of chronic diseases with increasing age. The necessity to maintain health and to invest in health capital increases with age. Therefore, the demand for GP visits and also prevention activities such as cervical cancer screening should increase. On the other hand, older women have a shorter life span and the pay-off period for preventative activities would be shorter. Also women of the highest age groups could be less able to visit their GP, because of reduced mobility as a consequence of physical handicaps. Another possibility could be that women of the higher age group more often visit the GP because of existing chronic conditions and have less time to focus on prevention such as a cervical cancer screening examination, because the focus of her GP visit is on the treatment of her chronic conditions. As a consequence, the effect of increasing age on the probability of visiting a GP cannot be predicted with confidence. The NHSCSP programme gives explicit rules how often screening examinations should be done at a certain age. Cervical cancer screening examination uptake should be higher for these age groups in comparison to other age groups. Empirical studies have found that the uptake rate of cervical cancer screening examination is highest in the recommended age interval [14–16]. Poor general health status which can be caused by chronic conditions should lead to an increased probability of a GP visit [10] and this hypothesis is empirically confirmed [17]. There could be less time and effort taken in doing the cervical cancer screening examination, because of the necessity to find the reason for the poor health status and the time consuming task to treat acute and chronic diseases as a priority. A higher educational level should lead to an increased uptake of a GP visit and cervical cancer screening examination, because women with a higher education level have a higher knowledge about health in general and about the importance of prevention including cervical cancer screening examinations in particular [14, 18]. A higher household income leads to higher household resources with an increased demand for time in perfect health. Therefore, the probability of a GP visit and the uptake of cervical cancer screening examinations should increase [9, 11]. The effect of an increasing household income on the probability of a GP visit and the uptake of cancer screening examinations was confirmed in three studies [10, 19, 20]. However, the effect of an increasing household income should be weaker in the UK in comparison to other countries, because GP visits and cervical cancer screening examinations are free of charge.

Non-economic factors such as socioeconomic determinants can also influence the probability of a GP visit and the uptake of the cervical cancer screening examination. Most of the empirical studies have neglected non-economic factors [21, 22] and the existing empirical literature is discussed in this and the following paragraph. The GP plays an important role as first point of contact in the UK health care system. The GP can give useful and important advice and information about the preventative importance of a cervical cancer screening examination. The GP can explain that regular cervical cancer screening examinations are essential for the early detection of cervical cancer. Uptake of cervical cancer screening examinations should be enhanced by a GP visit in the same period [23, 24]. Previous cervical cancer screening examinations have a predictive value for the uptake in the most recent period [14, 15, 21], because women are invited in certain time intervals for a screening examination. Smoking is correlated with more health risk taking behaviour and a weakened preference of individuals for health in comparison to non-health goods [25, 26] and women who smoke have poorer preventative health behaviour in general [27]. Smoking women have a lower probability of utilisation of healthcare services such a physician visits [28], and the predicted negative influence of smoking on screening examinations has been confirmed empirically [20, 29, 30]. An existing registration with a GP is a necessary condition to receive an invitation letter for the cervical cancer screening examination. Routine periodic invitation letters are sent from the GP practice according to the recommended NHSCSP interval. A woman lowers the chance of receiving an invitation letter if she has changed her address and residence. The uptake of cervical cancer screening examinations has been lower for women with a changed residence and address in one study [31], however not in another study [14].

The probability that a woman will visit a cervical cancer screening examination or a GP is also dependent on various further individual and household characteristics. Cohabitation status can be interpreted as an indicator for social support and a social network. Women who live in a partnership have better possibilities to exchange information about health promoting behaviour than non-cohabitating women [32, 33]. A higher number of children in the household could act as possible time constraint for GP visits and cervical cancer screening examinations. Two studies from European countries found that women with a higher number of children attended cervical cancer screening examinations less often [14, 31]. Women who work part-time or full-time may have higher opportunity costs for a GP visit or a cervical cancer screening examination in comparison to non-working and retired women. However, the empirical evidence was mixed in a systematic review which investigated the influence of employment on the cervical cancer screening uptake [21]. Non-white women could face emotional and cultural barriers for the cervical cancer screening examination, because a cervical cancer screening examination by a physician can be experienced as an invasive medical procedure. Ethnicity was the most important predictor for the probability of taking part in a cervical cancer screening examination and white British women had a higher uptake than women of other ethnicity [34].

Previous existing research with the British Household Panel Survey (BHPS) had analysed the uptake for cervical cancer screening examinations in two studies: Sabates et al. (2006) analysed the uptake with an unbalanced panel until 2003 [14] and Labeit et al. (2013) analysed the uptake with a balanced panel until 2008 [30] with a focus on previous screening history and other health related variables. Polisson (2011) analysed the uptake of GP visits in England using the General Household Survey (GHS) as a pseudo-panel: GP visit rates are determined by health status, i.e. existence of chronic diseases, and for women, by pregnancy and childbirth, i.e. women in their childbearing years had a higher probability of a GP visit than older women [17]. However, all mentioned empirical analyses in Great Britain had analysed the probability of a GP visit and a cervical cancer screening examination only separately.

There is no analysis which investigates if any determinants exist which increase or decrease both the probability of a GP visit and a cervical cancer screening examination in one year and if socioeconomic determinants exist which have a different effect on both uptakes. The following empirical analysis is based on a human capital approach with the inclusion of age, education, household income and health status as economic determinants and with the additional inclusion of non-economic factors, because non-economic factors can also play a decisive role for the uptake of GP visits and the cervical screening examination. The analysis compares the determinants of a cervical cancer screening examination with the determinants of a GP visit and additionally investigates if uptake of cervical screening examinations is more likely for women who visit their GP. It could potentially identify certain determinants which lead to a low probability of a GP visit and a preventive health check-up such as a cervical cancer screening examination and these determinants could be targeted by policy makers.

## Methods

The appropriate model would be a two-stage decision process model if the GP would be in a pure gate-keeper position for the cervical cancer screening examination. Such a model determines in a first step the factors of a GP visit and then in a second step the factors of a cervical cancer screening examination [16]. Such a two-stage decision process model could be modelled with a sample selection model [10]. A cervical cancer screening examination can be done within a GP practice in Great Britain or it can be done without visiting a GP practice by visiting a specialized service such as a family clinic or a genito-urinary medicine clinic. As a result, it is possible of getting a cervical cancer screening examination without visiting a GP practice and the GP it is not in a pure gate-keeper position. GP visits are not essential for the provision of cervical cancer screening examinations, but they are one possible determinant for the uptake of a cervical cancer screening examination. Therefore, the corresponding binary variable (GP visit) is included as explaining variable in the prevention equation. An appropriate statistical model should explain jointly the determinants of the cervical cancer examination and the determinants of the GP visit in one year and should consider also the endogeneity of the binary endogenous variable GP visit and the presence of non-observable factors. The recursive bivariate probit model is such a statistical model, because it allows for the estimation of the effect that a binary endogenous variable has on a binary outcome in the presence of unobservable variables [35]. Non-observable variables could be the women's anxiety and fear or the level of risk aversion and both variables can have an influence on the use of healthcare services in general [16]. The statistical model is presented in Eqs (1) and (2):

$$y_{GPi}^{*}=\alpha_{1i}+x_{i}^{'}\beta_{GPi}+\varepsilon_{GPi}$$ (1)

$$y_{CEi}^{*}=\alpha_{2i}+x_{i}^{'}\beta_{CEi}+\gamma GP+\varepsilon_{CEi}$$ (2)

In Eqs (1) and (2) α’s and ß’s and γ are parameter vectors which have to be estimated and *ε*_*GPi*_ and *ε*_*CEi*_ are the error terms of the GP and cervical cancer equation with the assumption of following a bivariate normal distribution. Individual and household socioeconomic characteristics are considered as covariates in both equations. The binary variable of a GP visit is included in the prevention equation, because the aim is to analyse the importance of the GP in prevention use. The preventive use of a cervical cancer screening examination is not included in the GP equation, because cervical cancer screening can be obtained without visiting a GP at other medical institutions. The estimation of the likelihood function can be done for the recursive bivariate probit model exactly in the same way as for the one of the normal bivariate probit model [36]. The endogeneity of the GP visit variable in the cervical cancer screening equation can be ignored for the estimation, because the likelihood function of the recursive bivariate probit model has the same form as the one of the regular bivariate probit model [36]. No exclusion restriction is necessary for the identification of the GP equation in the recursive bivariate probit model, however adding an exclusion restriction can increase the efficiency of the estimation. The lagged dependent variables are used as exclusion restrictions for the cervical cancer screening equation.

The screening guidelines of the NHSCSP and past participation in cervical cancer screening examinations are relevant for the uptake: it is sensible to consider the past screening behaviour, because there is an increased likelihood of participating in a screening examination after the recommended time interval of 3 years. Additionally, there exists the possibility that screening examinations are done more frequently, because there was an unclear test result in the previous year. Another possibility would be that a woman belongs to a high risk population with close relatives who have a history of cervical cancer. Lagged dependent variables up to order 3 were used.

## Data

The BHPS has information about GP visits and the uptake of cervical cancer screening examinations over a period of 17 years (1992 to 2008). The BHPS is a nationally representative sample of more than 5,000 households and all interviewed individuals within a household have to be 16 and older [37]. The first wave of the BHPS was in 1991. Questions about visiting a GP and participating in cervical cancer screening have been in every wave. For the analysis, an unbalanced sample of women from England, Scotland and Wales has been selected. Women from Northern Ireland have not been included in the analysis, because data collection has been started in Northern Ireland beginning from wave 11. The analysis has used the information from the period from 1992 to 2008 and so information for 17 waves is available.

Female individuals with private provision or with NHS and private provision for cervical cancer screening examinations have been excluded from the analysis and only women with NHS provision have been included. The dependent variable has taken the value of 1 for a specific panel year if a cervical cancer screening examination was done and 0 if no cervical cancer screening examination was done. There has been a policy change of the NHSCSP in 2003 and a dummy coding was chosen for analysing this policy change: women belonging to age group 25–49 for all years before and including the year 2003 were coded with 0 and women belonging to age group 25–49 for all the following years after the year 2003 with 1. The unbalanced panel for cervical cancer screening consisted of 8,386 women with 52,551 observations. The age categorisation has been according to the NHSCSP screening guidelines for the cervical cancer screening examination: 16 to 19 (reference category), 20 to 24, 25 to 49, 50 to 64, 65 and older. The age categorisation has been for a GP visit: 16 to 29 (reference category), 30 to 39, 40 to 49, 50 to 59, 60 to 69, 70 to 79 and 80 and above. The household income was defined as the total equivalised and deflated household annual income using the modified OECD scale to adjust for household size and needs [38]. The International Standard Classification of Education (ISCED) was used for the categorisation of educational levels. Different coded levels of education were tertiary, secondary and primary education level (reference category). Health status was self-rated and included as a variable with excellent (1) as reference category and good (2), fair (3), poor (4) and very poor (5) as further categories [39].

## Results

The recursive probit model is estimated for cervical cancer screening with lagged dependent variables as explaining variables and also with the inclusion of the GP visit variable in the same year.

Table 1 shows the proportion of women who have made a GP visit or a cervical cancer screening examination in the period between 1992 and 2008. The average rate for a GP visit was 81.84% per year over the whole observation period and 22.44% per year for a cervical cancer screening examination over the whole period. Participation rates for the cervical cancer screening examination decreased over the period.

**Table 1:** Uptake rate for a GP visit and cervical cancer screening examination during the 17 years period from 1992 to 2008 in Great Britain.

	GP visit	Cervical cancer screening
1992	80.50%	25.76%
1993	81.66%	25.82%
1994	81.71%	24.21%
1995	82.79%	24.79%
1996	82.51%	24.76%
1997	82.96%	24.17%
1998	84.12%	23.74%
1999	81.12%	21.56%
2000	82.64%	23.46%
2001	82.10%	23.56%
2002	82.25%	23.90%
2003	82.06%	22.43%
2004	80.19%	20.14%
2005	81.29%	20.20%
2006	80.97%	19.63%
2007	80.77%	18.98%
2008	82.39%	19.60%
Total	81.84%	22.44%

Source: BHPS. The unbalanced panels consisted for cervical cancer screening and GP visits of 8,386 women from 52,551 observations.

Table 2 gives descriptive statistics of the unbalanced panel for the GP and cervical cancer screening examination visit. Table 3 presents the result of the recursive probit model and the univariate models of the cervical cancer screening examination equation and the GP visit equation.

**Table 2:** Sample characteristics for the balanced sample of women from 1992 to 2008 in Great Britain.

	Frequency or mean/SD
Cervical cancer health check-up in period t	0.216
Cervical cancer screening examination 1 year before (t-1)	0.221
Cervical cancer screening examination 2 year before (t-2)	0.228
Cervical cancer screening examination 3 year before (t-3)	0.237
GP visit during last 12 months	0.816
Health status good	0.456
Health status fair	0.234
Health status poor	0.0891
Health status very poor	0.0263
Status smoking	0.225
Living with a partner	0.645
Number of children in household	0.600/(0.974)
Secondary education (ISCED)	0.407
Tertiary education (ISCED)	0.310
Moved residence within UK	0.0698
Age	49.686/(18.580)
Total equivalised and deflated HH annual income/100	3.005/(1.857)
Employed part-time or full-time	0.524
Region Scotland	0.140
Region Wales	0.120
Ethnic non-white	0.0198
Cervical screening policy change: year after 2003 and age group 25–49	0.197

Source: BHPS. The unbalanced panels consisted for cervical cancer screening and GP visits of 8,386 women from 52,551 observations.

**Table 3:** Univariate probit and recursive probit estimates of cervical cancer screening and GP visits in Great Britain.

**Prevention equation cervical cancer screening**	**Univariate probit cervical cancer**	**Univariate probit GP visit**	**Recursive probit**
Cervical cancer screening examination 1 year before (t-1)	0.445*** (0.0165)		0.444*** (0.0166)
Cervical cancer screening examination 2 years before (t-2)	-0.0491*** (0.0170)		-0.0492*** (0.0170)
Cervical cancer screening examination 3 years before (t-3)	0.661*** (0.0169)		0.660*** (0.0170)
GP visit during last 12months	0.368*** (0.0203)		0.478*** (0.129)
Healthstatus good	-0.0171 (0.0179)		-0.0344 (0.0272)
Healthstatus fair	-0.0158 (0.0217)		-0.0463 (0.0418)
Healthstatus poor	-0.0233 (0.0307)		-0.0592 (0.0521)
Healthstatus very poor	0.0395 (0.0509)		0.00199 (0.0671)
Smoking	0.0461*** (0.0175)		0.0494*** (0.0180)
Household income	0.00528 (0.00399)		0.00512 (0.00399)
Living with a partner	0.0392** (0.0176)		0.0384** (0.0176)
Number of children in household	0.0203***(0.00781)		0.0212***(0.00789)
Secondary education (ISCED)	0.0390* (0.0222)		0.0366 (0.0224)
Tertiary education (ISCED)	0.0658*** (0.0237)		0.0628*** (0.0240)
Employed	0.0509*** (0.0178)		0.0539*** (0.0182)
Moved residence	0.0377 (0.0253)		0.0336 (0.0258)
Region Scotland	-0.0239 (0.0196)		-0.0250 (0.0196)
Region Wales	0.00707 (0.0223)		0.00775 (0.0223)
Race non-white	-0.0826* (0.0453)		-0.0873* (0.0457)
Age 20–24	0.425*** (0.0407)		0.418*** (0.0416)
Age 25–49	0.343*** (0.0326)		0.342*** (0.0326)
Age 50–64	0.150***´(0.0310)		0.152*** (0.0311)
Age 65 and older	-0.903*** (0.0376)		-0.903*** (0.0376)
After year 2003xAge 25–49	-0.0377** (0.0171)		-0.0370** (0.0171)
Constant	-1.632*** (0.0394)		-1.700*** (0.0874)
**GP visit equation**		**Univariate probit GP visit**	**Recursive probit**
Healthstatus good		0.490*** (0.0203)	0.490*** (0.0203)
Healthstatus fair		1.053*** (0.0278)	1.053*** (0.0278)
Healthstatus poor		1.462*** (0.0427)	1.463*** (0.0427)
Healthstatus very poor		1.687*** (0.0780)	1.687*** (0.0780)
Smoking		-0.136*** (0.0264)	-0.136*** (0.0264)
Household income		0.00497 (0.00575)	0.00498 (0.00574)
Living with a partner		0.0466* (0.0243)	0.0463* (0.0243)
Number of children in household		-0.0419*** (0.0124)	-0.0419*** (0.0124)
Secondary education (ISCED)		0.0751** (0.0306)	0.0751** (0.0305)
Tertiary education (ISCED)		0.0949*** (0.0330)	0.0948*** (0.0330)
Employed		-0.104*** (0.0257)	-0.105*** (0.0257)
Moved residence		0.111*** (0.0284)	0.110*** (0.0284)
Region Scotland		0.0466 (0.0302)	0.0463 (0.0302)
Region Wales		-0.0272 (0.0333)	-0.0267 (0.0334)
Race non-white		0.220*** (0.0798)	0.220*** (0.0798)
Age 30–39		-0.187*** (0.0328)	-0.187*** (0.0328)
Age 40–49		-0.380*** (0.0349)	-0.381*** (0.0349)
Age 50–59		-0.346*** (0.0388)	-0.347*** (0.0388)
Age 60–69		-0.285*** (0.0430)	-0.283*** (0.0433)
Age 70–79		-0.196*** (0.0477)	-0.198*** (0.0481)
Age 80 and above		-0.243*** (0.0577)	-0.246*** (0.0583)
Constant	-1.632*** (0.0394)	0.593*** (0.0509)	0.594*** (0.0510)

Source: BHPS. Unbalanced panels consisted for cervical cancer screening of 8,386 women from 52,551 observations. Robust SEs are displayed in parentheses, to account for individual repeated observations in the panel.

*p<0.1;

**p<0.05;

***p<0.01.

Living with a partner, a higher education, women of non-white race, a worsening self-perceived health status and a relocated residence led to a higher probability of visiting a GP. In contrast, employed women, smoking women and women with a higher number of kids had a lower probability for a GP visit. Women of the reference age group (age 16 to 29) have the highest probability of a GP visit, followed by a decrease and a second peak for the age group 70 to 79. This second peak is followed by a decline for the oldest age group.

Taking part in a cervical cancer screening examination one year and three years earlier showed a strong positive influence on the current uptake of a screening examination. A GP visit had a positive influence on the probability of doing a cervical cancer screening examination in the same year and also living with a partner, a higher number of kids, being employed, tertiary education and smoking. Women of non-white race had a lower probability of doing a cervical cancer screening examination. Women in the recommended age groups for cervical cancer screening examination had an increased uptake for cervical cancer screening examination in comparison to the youngest and oldest age group. Women with an age between 25 and 49 had no increased uptake of cervical cancer screening examinations after the change of the NHSCSP guidelines in 2003.

## Discussion

This empirical analysis investigated for the first time the joint probability of a GP visit and cervical cancer screening examination in Great Britain. As econometric estimation method a recursive probit model was used and the time period between 1992 and 2008 was analysed. The empirical investigation showed that only two variables, education and living with a partner, increased the probability of visiting a GP and a cervical screening examination. All other analysed variables had a different influence on both probabilities.

A GP visit in the same year as a cervical cancer screening examination leads to a higher uptake of cervical cancer screening examinations. This result is in accordance with the hypothesis that the GP plays an important role as gatekeeper in the UK health care system and the GP gives advice about prevention which includes cervical cancer screening and the cervical cancer screening examination can also be done by GP [24]. The results are similar to those of an Italian study which has analysed the uptake of cervical cancer screening examinations with a recursive probit, because GP visits have led in both studies to an increased uptake of cervical screening examinations [16]. The cervical cancer screening examination can be done in both countries at a GP practice. However, a visit of a GP practice is in both countries not an essential condition for the provision of a cervical cancer screening examination and the examinations can also be done in specialized services such as family clinic or the genito-urinary medicine (GUM) department of a hospital.

Health status can be interpreted as a proxy for health and so a poor self-perceived health status increased the probability of visiting a GP and this result is in accordance with the Grossman model [9]: the higher probability can be explained by the fact that women in a poor self-assessed health status could have a high demand of investing in their health and investing in the health stock is necessary because of the treatment of acute and chronic diseases. The non-significant effect of health status on the cervical cancer screening examination in contrast to the effect on a GP visit is in accordance with this explanation, because treatment of the acute and chronic medical conditions is a competing health priority which comes first. However, poor self-assessed health status can influence uptake also in other ways and these reasons could also prevent women from taking part in cervical cancer screening examinations. Firstly, there could be lower perceptions on the preventability of health problems and all types of diseases and cancer. Secondly, women with poorer health status could also express less interest in receiving prevention information about cervical cancer [40]. Thirdly, psychological factors such as fear and anxiety about confirmation of cervical cancer could be related to a poor health status. Smoking had a negative impact on the probability of visiting a GP, but a positive one on visiting a cervical cancer screening examination [16]. The first result could be interpreted as confirmation of smoking as an indicator for health risk taking behaviour and smokers have shown a reduced healthcare utilisation including physician visits [25, 28], however not the second result could be explained in this way. There could exist a reverse causality of cervical screening examinations on smoking behaviour. Women who report current and recent smoking have an increased likelihood of cervical abnormalities with higher probabilities of positive smear test results in screening examinations [41]. An increased probability of further cervical cancer screening examinations could follow. A positive test result could lead women to give up their smoking, because these women want to change their health behaviour and become more health-conscious and these women would have less cervical abnormalities in the future. As a further consequence, non-smoking women could have a decreased probability of cervical cancer screening examinations. The strong influence of past screening behaviour with a positive significant effect of the own first order lag and the third order lag cervical cancer screening shows that past screening behaviour influences the behaviour in the most recent period. This result can be interpreted as persistence in screening behaviour or state dependence [42]. The NHSCSP gives explicit rules for the time interval between screening examinations. The importance of these screening guidelines on current behaviour can be seen especially in the high predictive value of a cervical cancer screening examination which has been done three years before. Also the coefficient for a screening examination which has been done one year before is positive. The coefficient for the first order lag could be explained by the possibility that a necessary control follow-up is necessary to check an unclear test result from a previous cervical cancer screening examination.

When using a recursive probit model, it is not possible to differentiate between unobserved heterogeneity and state dependence. For differentiating between these two econometric possibilities it would be necessary to use a dynamic panel probit model with a Wooldridge-type estimator. It has been shown that unobserved heterogeneity can play an important role in explaining variation [30]. A further weakness in the used recursive probit model lies in the fact that the lagged dependent variables are assumed to be exogenous and not endogenous such as in a dynamic panel data model.

The relevance of the strict age recommendations of the NHSCSP can be seen in the specifications with the highest probability of uptake in the recommended age groups. The finding of a lower screening uptake in the oldest age group in comparison to younger age groups can be explained with the shorter pay-off period for older women and the lower incidence of cervical cancer in older age groups [43]. The change of the cervical cancer screening policy in 2003 for the age group 25 to 49 with shortening the recommended time interval from 5 to 3 years had no effect. The reason why the policy change in cervical cancer screening had no effect could be based on the fact that before 2003 85% of the PCT’s decided themselves for a 3-year invitation policy [7]. The result of the relationship between age and the likelihood of a GP visit shows that women with age 30 and below have a higher probability of visiting a GP than women of the adjacent older age groups. The probability of a GP visit increases again for the higher age groups and then it declines for the oldest age group. The present analysis is in accordance with an analysis which investigates the probability and number of GP visits by sex and age in England [17]. This analysis has shown that the probability and number of GP visits have a first peak around age 30 and then it is followed by a decline and then a second peak and it also confirms the present analysis that the probability of a GP visit for women is most driven by the health status and age of the women.

In two systematic reviews which has analysed the determinants of screening uptake for different cancer screening examinations, none of the analysed socioeconomic variables has been significant in all analysed studies [21, 22]. The estimations confirm the result of these systematic reviews, because different socioeconomic variables are of importance for the GP visit and the cervical cancer screening examination. Only secondary and tertiary education and living with a partner has increased the probability in both equations. Results are in accordance with the prediction, because higher education leads through different influence channels to an increased demand for health care such as GP visits and prevention. Living within a partnership can be interpreted as an indicator for a better social network. A functioning social network could increase the propensity of a woman to visit her GP visit and to take part in preventative screening examinations. Other socioeconomic variables have had inconsistent results in both equations. A higher number of children and being employed has decreased the probability of visiting a GP, but has increased the possibility of having a cervical cancer screening examination. Non-white women have had a higher probability of visiting a GP which is in accordance with another empirical analysis for England [44], but a lower probability of having a cervical cancer screening examination. Most of the non-white women are of black and Asian ethic origin and cultural and emotional barriers exist in taking part in a cervical cancer screening examination and the examination can be perceived by women as an invasive intimidating procedure [45]. Lack of awareness and low perceived risk are also reasons for the lower cervical screening coverage [45].

A first limitation exists, because there is no information about the location of the screening unit available, i.e. there is no information available on whether the cervical screening examination has been done in a GP practice or in another location. Characteristics of the screening unit such as structure and organization of medical services performing the screening test can influence the uptake rate. Such an association has been shown for cervical cancer screening uptake and GP practice characteristics in England [46]. A second limitation of the analysis exists, because there is no information about results from previous smear tests available. Therefore, it is not possible to make a distinction between a regular preventative and a control follow-up cervical screening examination, which is done in response to previous inconclusive results. A third limitation is that no personal or family history of cervical cancer is available in the dataset. It is recommended to do a smear test annually for these women and these women would probably have a higher uptake [47]. A fourth limitation exists, because there is no information about the level of trust in the GP or in the NHS available, because it has been shown that a visit of a GP or doing a cervical cancer screening examination can be dependent on the trust in these institutions [16]. A fifth limitation exists, because a recursive probit model considers no unobserved heterogeneity and lagged dependent variables are assumed as exogenous.

## Conclusions

The innovative feature is to analyse the determinants of a GP visit and cervical cancer screening examination with a recursive panel probit model for Great Britain. There is no analysis until now that investigates which determinants are common and which are different for a GP visit and a cervical cancer screening examination. The analysis shows that only higher education and living with a partner increases both probabilities and a poor health status increases only the probability of a GP visit, but not for the cervical cancer screening examination. Also the influence of a GP visit on the probability of a cervical cancer screening examination is important. No determinants could be identified which lead both to a low probability of a GP visit and a cervical cancer screening examination and could be easily targeted.

An implication for policymakers and practitioners is that a GP visit promotes the uptake of the cervical cancer screening examination and his role in preventive health check-ups such as a cervical cancer screening examination should not weakened. Further research would be necessary to analyse how a cervical cancer screening examination competes with other medical activities such as the time intensive treatment of acute and chronic disease conditions at a GP visit and how this could affect preventive screening examinations during a GP visit.
